# Impact of contact resistance on the electrical properties of MoS_2_ transistors at practical operating temperatures

**DOI:** 10.3762/bjnano.8.28

**Published:** 2017-01-25

**Authors:** Filippo Giannazzo, Gabriele Fisichella, Aurora Piazza, Salvatore Di Franco, Giuseppe Greco, Simonpietro Agnello, Fabrizio Roccaforte

**Affiliations:** 1 Consiglio Nazionale delle Ricerche – Istituto per la Microelettronica e Microsistemi, Strada VIII, n 5, 95121 Catania, Italy; 2 Department of Physics and Chemistry, University of Palermo, Via Archirafi 36, 90143 Palermo, Italy; 3 Department of Physics and Astronomy, University of Catania, Via Santa Sofia, 64, 95123 Catania, Italy

**Keywords:** contact resistance, mobility, MoS_2_, temperature dependence, threshold voltage

## Abstract

Molybdenum disulphide (MoS_2_) is currently regarded as a promising material for the next generation of electronic and optoelectronic devices. However, several issues need to be addressed to fully exploit its potential for field effect transistor (FET) applications. In this context, the contact resistance, *R*_C_, associated with the Schottky barrier between source/drain metals and MoS_2_ currently represents one of the main limiting factors for suitable device performance. Furthermore, to gain a deeper understanding of MoS_2_ FETs under practical operating conditions, it is necessary to investigate the temperature dependence of the main electrical parameters, such as the field effect mobility (μ) and the threshold voltage (*V*_th_). This paper reports a detailed electrical characterization of back-gated multilayer MoS_2_ transistors with Ni source/drain contacts at temperatures from *T* = 298 to 373 K, i.e., the expected range for transistor operation in circuits/systems, considering heating effects due to inefficient power dissipation. From the analysis of the transfer characteristics (*I*_D_−*V*_G_) in the subthreshold regime, the Schottky barrier height (Φ_B_ ≈ 0.18 eV) associated with the Ni/MoS_2_ contact was evaluated. The resulting contact resistance in the on-state (electron accumulation in the channel) was also determined and it was found to increase with *T* as *R*_C_ proportional to *T*^3.1^. The contribution of *R*_C_ to the extraction of μ and *V*_th_ was evaluated, showing a more than 10% underestimation of μ when the effect of *R*_C_ is neglected, whereas the effect on *V*_th_ is less significant. The temperature dependence of μ and *V*_th_ was also investigated. A decrease of μ proportional to 1/*T*^α^ with α = 1.4 ± 0.3 was found, indicating scattering by optical phonons as the main limiting mechanism for mobility above room temperature. The value of *V*_th_ showed a large negative shift (about 6 V) increasing the temperature from 298 to 373 K, which was explained in terms of electron trapping at MoS_2_/SiO_2_ interface states.

## Introduction

Transition metal dichalcogenides (TMDs) are compound materials formed by the Van der Waals stacking of MX_2_ layers (where M = Mo, W, etc., i.e., a transition metal, and X = S, Se, Te, i.e., a chalcogen atom). Among the large number of existing layered materials [1], TMDs are currently attracting increasing scientific interest due to some distinct properties, such as the presence of a sizable bandgap in their band structure. As an example, MoS_2_ (the most studied among TMDs due to its high abundance in nature and relatively high stability under ambient conditions) exhibits an indirect bandgap of ≈1.3 eV in the case of few layers and bulk material and a direct bandgap of ≈1.8 eV in the case of a single layer. These properties make MoS_2_ an interesting material for the next generation of electronics and optoelectronics devices [2]. As an example, field effect transistors with very interesting performance in terms of the on/off current ratio (10^6^–10^8^) and low subthreshold swing (≈70 meV/decade) have been demonstrated using single [3] and multilayers of MoS_2_ [4].

MoS_2_ thin films, obtained either by cleavage from the bulk material or by chemical vapor deposition, are typically unintentionally n-type doped. Since well-assessed methods for doping enrichment of MoS_2_ under source/drain contacts are still lacking, MoS_2_ transistors are mostly fabricated by deposition of metals directly on the unintentionally doped material, resulting in the formation of Schottky contacts. Experimental investigations showed that both low work function (e.g., Sc, Ti) and high work function (e.g., Ni, Pt) metals mostly exhibit a Fermi level pinning close to the conduction band of MoS_2_ [5], resulting in a Schottky barrier height (SBH) for electrons typically ranging from 0.1 to 0.3 eV. The origin of this Fermi level pinning is currently a matter of investigation and a crucial role seems to be played by nanoscale defects/inhomogeneities at the metal/MoS_2_ interface [6–7].

The presence of this small but not negligible Schottky barrier at source/drain contacts certainly has a strong impact on the electrical characteristics of MoS_2_ transistors in the subthreshold regime [5]. In addition, the resulting source/drain contact resistance, *R*_C_, can also have a significant influence on the electrical properties of the device in the on-state, i.e., above the threshold voltage (*V*_th_). In particular, *R*_C_ is expected to affect, to some extent, the values of *V*_th_ and of the field effect mobility μ extracted from the transfer characteristics (drain current, *I*_D,_ vs gate bias, *V*_G_) of the device and of the on-resistance (*R*_on_) extracted from the output characteristics (drain current, *I*_D,_ vs drain bias, *V*_DS_). Clearly, all these parameters (*V*_th_, μ, and *R*_on_) have their own dependence on the temperature, and their combination results in the device electrical characteristics at a fixed measurement condition. Hence, to gain a deeper understanding of the behavior of MoS_2_ transistors for real applications, a temperature-dependent characterization of the main electrical parameters under practical operating conditions is mandatory. A temperature range from room temperature to 400 K is a realistic range for device operation in circuits/systems, taking into account the heating effect they undergo due to inefficient heat dissipation. However, to date, only a limited number of papers have focused on the high temperature behavior of MoS_2_ transistors [8–9].

In this paper, we report a detailed temperature dependent investigation of multilayer MoS_2_ transistors with Ni source/drain contacts, focusing on the role played by the contact both in the subthreshold regime and above the threshold voltage. In contrast to other literature works, mainly focused on the use of low work function contacts (such as Sc or Ti) to minimize the effect of contact resistance in n-type MoS_2_ FETs [5], we focused on a high work function metal such as Ni in this paper in order to evaluate the impact of Ni/MoS_2_ contact resistance on the device field effect mobility μ and threshold voltage *V*_th_. The interest on Ni was also motivated by the recently demonstrated possibility to achieve MoS_2_ FETs with ambipolar behavior by performing a temperature-bias annealing processes on as-deposited Ni contacts [10]. In the following, the temperature dependence of μ, *V*_th_ and *R*_C_ in the range from 298 to 373 K was determined and the physical mechanisms of these dependences were discussed.

## Results and Discussion

Back-gated transistors have been fabricated using multilayer MoS_2_ flakes (with thickness ranging from ≈40 to ≈50 nm) exfoliated from bulk molybdenite crystals onto a highly doped Si substrate covered with 380 nm thick, thermally grown SiO_2_. Such relatively thick MoS_2_ samples have been chosen since it has been reported that the electrical properties (μ, *V*_th_) of simple back-gated transistors fabricated with multilayer MoS_2_ are much less affected by the effect of the external environment (water/oxygen) [9] with respect to single or few layer devices [11], for which encapsulation is instead required to achieve good electrical performance [3].

Furthermore, as reported in the literature, carrier mobility is only slightly dependent on MoS_2_ thickness for transistors fabricated on ≈20 to ≈70 nm thick flakes, whereas stronger variations are observed for thinner flakes, with the largest mobility values obtained for thicknesses ranging from 6 to 12 nm [5].

**Figure 1:**
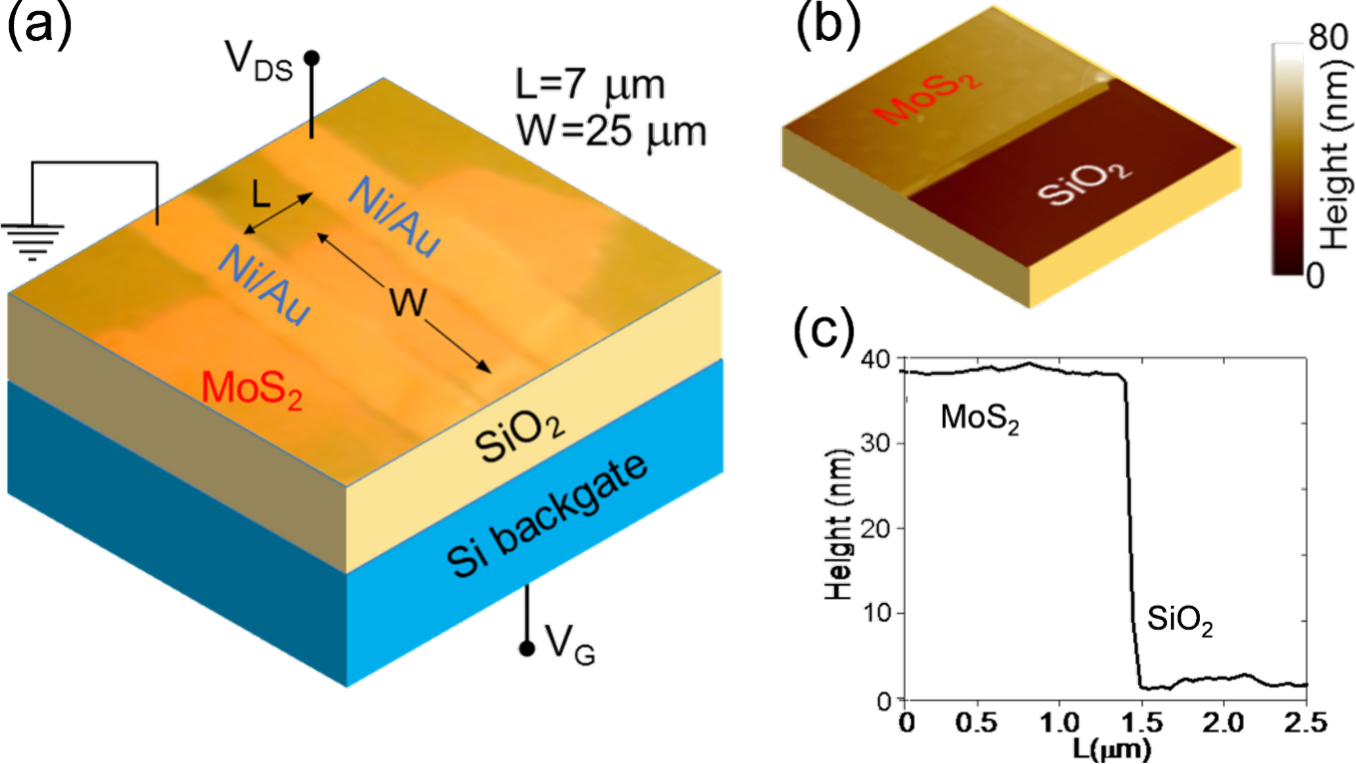
(a) Schematic including an optical image of a MoS_2_ transistor with the SiO_2_/Si backgate and Ni/Au source and drain contacts. (b) Atomic force microscopy image and (c) the corresponding height linescan of the MoS_2_ flake on the SiO_2_ substrate.

The experiments discussed in this paper have been carried out on a set of ten FETs fabricated on the same substrate. For consistency, the reported temperature-dependent analysis has been carried out on one of the transistors from this set of devices. Figure 1a shows a schematic representation including an optical image of a MoS_2_ transistor with the SiO_2_/Si backgate and Ni/Au source and drain contacts. An atomic force microscopy image (Figure 1b) and the corresponding height linescan (Figure 1c) of the MoS_2_ flake on the SiO_2_ substrate are also reported, showing ≈40 nm flake thickness.

**Figure 2:**
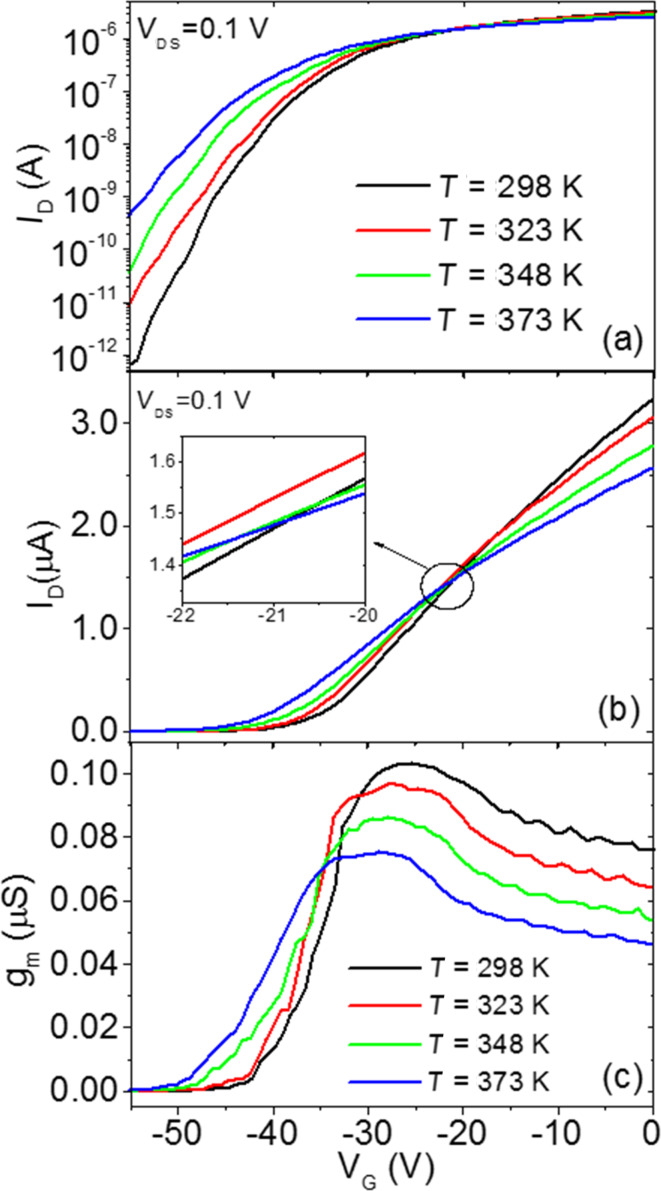
Semilog scale plot (a) and linear scale plot (b) of the transfer characteristics (*I*_D_−*V*_G_) measured at a fixed drain bias (*V*_DS_ = 0.1 V) and at different temperatures ranging from 298 to 373 K. (c) Transconductance (*g*_m_ = d*I*_D_/d*V*_G_) vs *V*_G_ curves at different temperatures calculated from the *I*_D_−*V*_G_ characteristics in (b).

The transfer characteristics (*I*_D_−*V*_G_) measured at a low fixed drain bias (*V*_DS_ = 0.1 V) on this device at different temperatures from 298 to 373 K are reported in Figure 2 both on a semilogarithmic scale (Figure 2a) and on a linear scale (Figure 2b). Clearly, the linear scale plot allows the current transport above the threshold voltage (*V*_th_) to be studied, whereas the semilog scale plot allows for a better visualization of transport in the subthreshold regime. In the following two sections, a detailed analysis of the characteristics in the subthreshold and above-threshold regime will be reported and the device electrical parameters will be extracted. In particular, the Ni/MoS_2_ Schottky barrier height and the flat band voltage (*V*_FB_) will be evaluated from the temperature-dependent analysis of the subthreshold *I*_D_−*V*_G_ curves, whereas the temperature behavior of *V*_th_ and μ will be obtained from the curves above the threshold.

### Subthreshold behavior

The semilog scale *I*_D_−*V*_G_ characteristics (Figure 2a) measured at 298 K exhibit a current variation of more than six orders of magnitude in the bias range from *V*_G_ = −55 V to 0 V. This current variation is significantly reduced with increasing the temperature from 298 to 373 K, especially due to the strong increase of current with the temperature at large negative bias.

**Figure 3:**
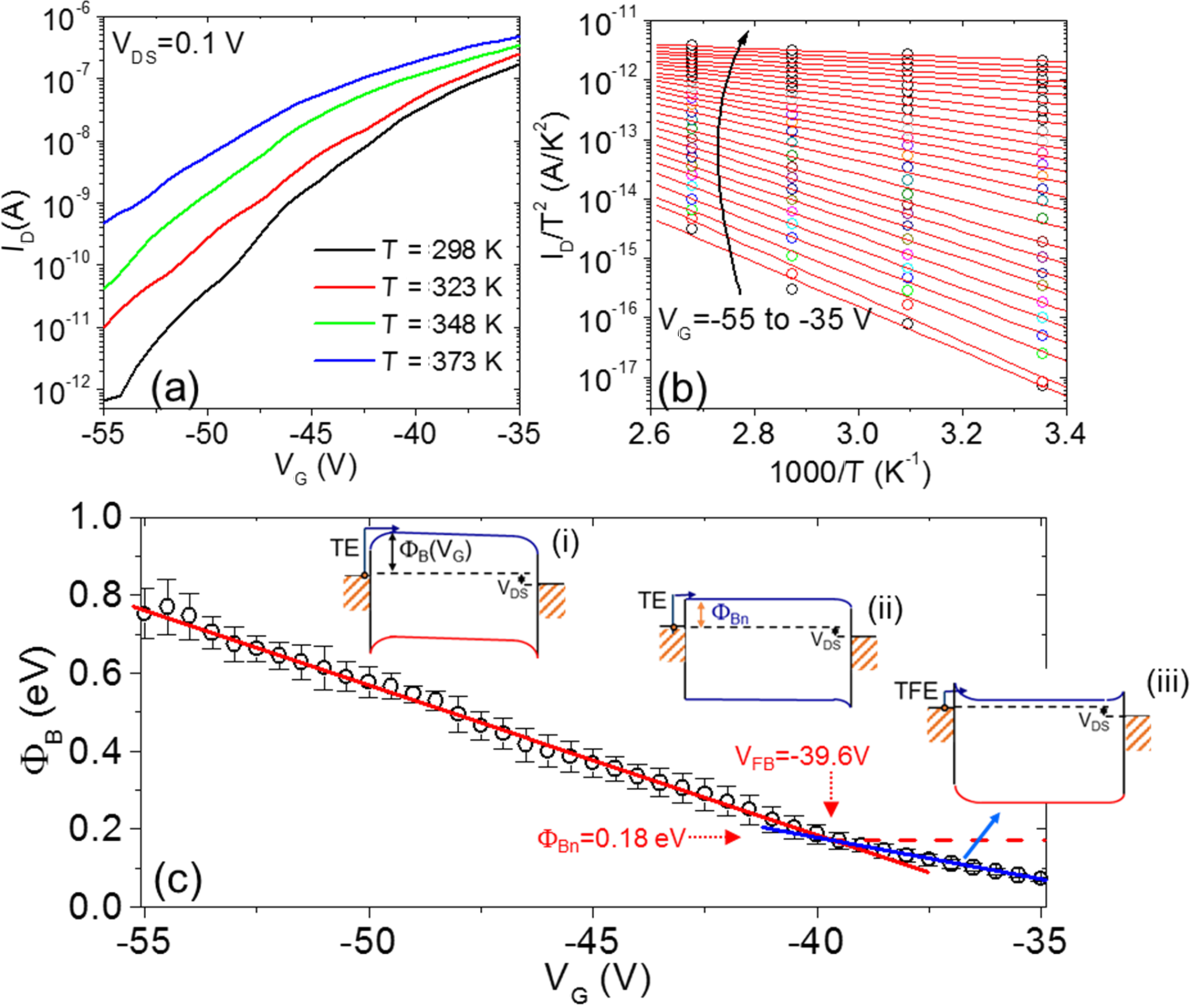
(a) Semilog scale plot of the transfer characteristics (*I*_D_−*V*_G_) in the subthreshold regime at different temperatures ranging from 298 to 373 K. (b) Arrhenius plot of *I*_D_/*T*^2^ vs 1000/*T* for each gate bias. (c) Effective SBH Φ_B_ as a function of *V*_G_. In the inserts, schematic band diagrams are given for the transistor operation in different regimes: depletion (i), flat band voltage (ii) and accumulation (iii).

This is better highlighted in Figure 3a, where the *I*_D_−*V*_G_ characteristics in the gate bias range from −55 to −35 V and at different temperatures from 298 to 373 K have been reported. Such strong dependence of *I*_D_ on *T* suggests that current transport in the subthreshold regime is dominated by thermionic current injection through the reverse biased source/MoS_2_ Schottky contact, according to the relation 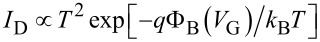 [5], where Φ_B_(*V*_G_) is the effective Schottky barrier height (SBH), modulated by the gate bias *V*_G_. To verify this, for each *V*_G_ an Arrhenius plot of *I*_D_/*T^2^* vs 1000/*T* is reported in Figure 3b. A nice linear dependence was observed for all the *V*_G_ in the considered bias range. The effective SBH values Φ_B_, obtained from the slope of the linear fit of the Arrhenius plot in Figure 3b are reported in Figure 3c as a function of *V*_G_. The schematic band diagrams corresponding to the different transistor operation regimes, i.e., depletion (i), flat band (ii) and accumulation (iii), are also illustrated in the inserts of Figure 3c.

In the depletion regime (Figure 3c (i)), the applied gate bias induces an upward band bending, ψ, in MoS_2_ at the interface with the SiO_2_ gate insulator. The experimentally evaluated SBH is found to depend linearly on *V*_G_. This dependence can be fitted with the relation 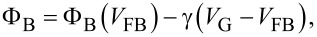 where Φ_B_(*V*_FB_) is the effective SBH at the flat band voltage and the term ψ = γ(*V*_G_−*V*_FB_) is the upward band bending. The slope γ indicates the modulation efficiency of Φ_B_ by the gate bias. It depends on the SiO_2_ layer capacitance, *C*_ox_ = ε_0_ε_ox_/*t*_ox_ ≈ 9.1 × 10^−5^ F/m^2^ (ε_0_ is the vacuum dielectric constant, ε_ox_ = 3.9, *t*_ox_ = 380 nm, the permittivity and the thickness of the SiO_2_ film, respectively), on the capacitance of the MoS_2_ depletion region, *C*_s_, as well as on the capacitance associated with MoS_2_/SiO_2_ interface traps, *C*_it_ [5]. In the depletion regime, the current transport in the transistor is ruled by thermionic emission (TE) of electrons from the source contact to the channel.

The effective SBH Φ_B_ and, hence, the band bending ψ = Φ_B_(*V*_G_)−Φ_B_(*V*_FB_) is found to decrease linearly moving toward positive *V*_G_ values. The flat band voltage *V*_FB_ corresponds to the gate bias for which ψ = 0 (see (ii) in Figure 3b), whereas for *V*_G_ > *V*_FB_ the band bending ψ < 0 (see (iii) in Figure 3b), i.e., the channel starts to accumulate electrons. In the accumulation regime, current injection in the channel is ruled by thermionic field emission (TFE) through the source triangular barrier. The TFE mechanism yields a reduced effective SBH with respect to the constant Φ_B_ value (red dashed line in Figure 3c) that would be expected if only TE over the barrier would occur. As a guide for the eye, the SBH dependence on *V*_G_ in the accumulation regime has been fitted with a blue line in Figure 3c. Hence, *V*_FB_ = −39.6 V can be experimentally determined as the bias corresponding to the intercept between the two linear fits [5]. The corresponding SBH value Φ_B_(*V*_FB_) = 0.18 eV represents the “real” (i.e., gate bias independent) value of the Ni/MoS_2_ Schottky barrier.

The experimental *V*_FB_ for the MoS_2_ transistor exhibits a large negative value, as reported in other literature works [12]. Such a result has been ascribed to donor-like interface trap states (positively charged when empty) between the SiO_2_ and MoS_2_ [13–14]. In order to evaluate the amount of this positive charge at the interface, it is worth comparing the experimental value with the one deduced from theoretical expression of the flat band voltage (*V*_FB,id_) of an ideal metal-oxide-semiconductor field effect transistor (i.e., without fixed or interface charges). *V*_FB,id_ is expressed as [15]:

[1]
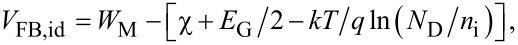


where *W*_M_ is the work function of the gate material (4.05 eV for the n^+^-doped Si back gate in our transistor), χ is the semiconductor electron affinity (4.2 eV for MoS_2_), *N*_D_ is the semiconductor doping concentration (on the order of 10^16^ cm^−3^ in unintentionally doped MoS_2_) and *n*_i_ is the intrinsic carrier concentration (for MoS_2_, 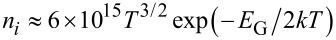 cm^−3^). According to this expression a low value of *V*_FB,id_ slightly varying with the *T* (from −0.35 V at 298 K to −0.42 V at 273 K) would be expected for our device. The negative shift of the experimental *V*_FB_ with respect to *V*_FB,id_ can be accounted for by the presence of a net positive charge density at the interface with SiO_2_ that can be evaluated as *C*_ox_(*V*_FB_−*V*_FB,id_)/*q* ≈ 2.2 × 10^12^ cm^−2^.

In the following section, the device transfer characteristics above the threshold will be analyzed to extract the threshold voltage and mobility.

### Transfer characteristics above threshold

The linear scale transfer characteristics (Figure 2b) show very low current below a threshold voltage (*V*_th_) and a nearly linear increase of *I*_D_ vs *V*_G_ above *V*_th_. Two effects can be observed from the comparison of the *I*_D_−*V*_G_ curves at increasing temperatures, i.e., (i) a negative shift of the threshold voltage and (ii) a decrease of the *I*_D_−*V*_G_ curve slope in the linear region above *V*_th_. The origin of these two effects will be discussed more in detail later on. Interestingly, as a result of these two competing effects, the *I*_D_−*V*_G_ characteristics tend to cross nearly at the same gate bias *V*_G_ = −21 V (see details in the insert of Figure 2b). This bias condition can be interesting for some applications where it is desirable that the device performance does not depend significantly on the temperature (d*I*_D_/d*T* ≈ 0).

Figure 2c shows the transconductance *g*_m_ vs *V*_G_ curves calculated by differentiation (*g*_m_ = d*I*_D_/d*V*_G_) of the *I*_D_−*V*_G_ characteristics in Figure 2b. In the considered bias range, all the curves exhibit an increase of *g*_m_ with *V*_G_ up to a maximum value, followed by a decrease of *g*_m_. The maximum transconductance value (*g*_m,max_) is found to decrease with increasing temperature. Furthermore, a rigid shift of the *g*_m_–*V*_G_ curves toward negative gate bias values is observed with increasing *T*.

**Figure 4:**
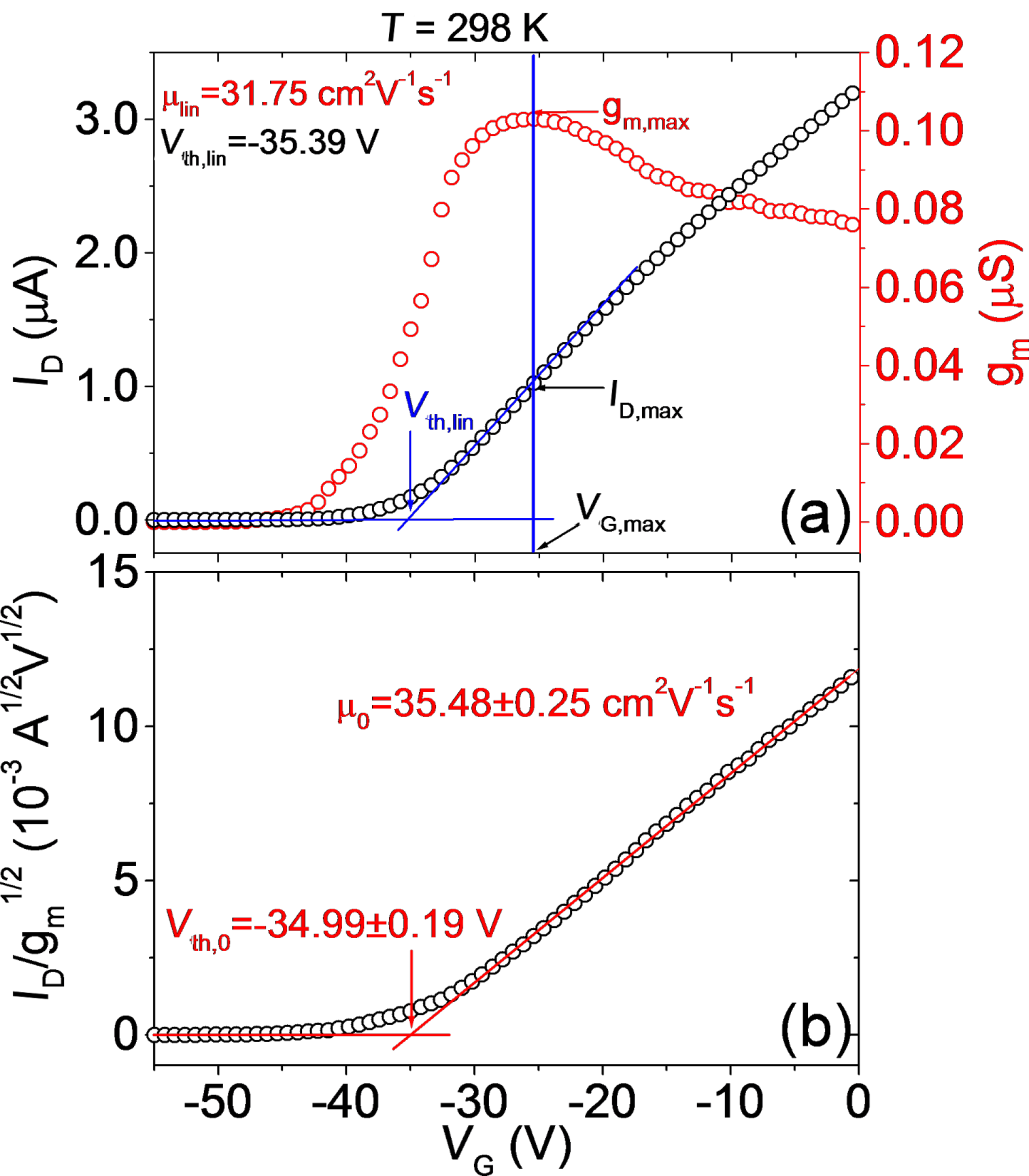
(a) Linear scale plot of *I*_D_ (left axis) and of the transconductance *g*_m_ (right axis) at *V*_DS_ = 0.1 V and *T* = 298 K. The extraction of the field effect mobility (μ_lin_) from the maximum of *g*_m_ and of *V*_th_ by the linear extrapolation method (*V*_th,lin_) is illustrated. (b) Plot of *I*_D_/*g*_m_^1/2^ vs *V*_G_ with illustration of extraction of the mobility (μ_0_) and threshold voltage value (*V*_th,0_) corrected by the effect of the contact resistance (*R*_C_).

From the linear scale transfer characteristics and the transconductance, two key electrical parameters for transistor operation, i.e., the threshold voltage (*V*_th_) and the field effect mobility (μ), are typically evaluated. Figure 4a shows a linear scale plot of *I*_D_ (left axis) and of the transconductance *g*_m_ (right axis) at *V*_DS_ = 0.1 V and *T* = 298 K. The field effect mobility in the linear region, μ_lin_, of the transfer characteristics is typically extracted from the transconductance using the following formula 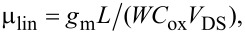 where *L* and *W* are the channel length and width, respectively, and *C*_ox_ the SiO_2_ gate capacitance. For our device with *L*/*W* = 7 μm/25 μm and *C*_ox_ ≈ 9.1 × 10^−5^ F/m^2^, the evaluated mobility from the maximum transconductance value *g*_m,max_ was μ_lin_ = 31.75 cm^2^V^−1^s^−1^, as indicated in Figure 4a. A method for evaluating *V*_th_ consists of drawing the tangent line to the *I*_D_−*V*_G_ curve at the bias (*V*_G,max_) corresponding to *g*_m,max_ and taking the intercept with the *I*_D_ = 0 baseline [16], as shown in Figure 4a.

This procedure can be explained by simple geometrical considerations. In the linear region of the transfer characteristics, *I*_D_ can be expressed as [14]:

[2]
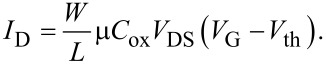


Hence, it results that *I*_D,max_ = *g*_m,max_(*V*_G,max_−*V*_th_) and the threshold voltage can be calculated as


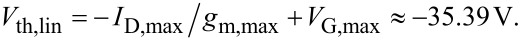


As a matter of fact, for the evaluation of *V*_th,lin_ and μ_lin_ based on Equation 2 the contribution of the contact resistance is assumed to be zero. However, as deduced from the analysis of the subthreshold characteristics, a Schottky barrier is associated to the source/drain contacts with MoS_2_, which is also expected to result in a non-negligible contact resistance, *R*_C_. The value of the contact resistance above the threshold and its temperature dependence will be estimated in the last section of this paper from the analysis of the on-resistance (*R*_on_) extracted from the device output characteristics (*I*_D_−*V*_DS_) at low *V*_DS_. Here, we want to discuss how *R*_C_ can influence the evaluation of μ and *V*_th_ from the transfer characteristics.

In order to take into account the role of *R*_C_, *V*_DS_ can be replaced by *V*_DS_−*I*_D_*R*_C_ in Equation 2, and solving by *I*_D_, the following expression for *I*_D_ is obtained:

[3]
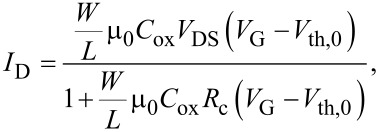


where μ_0_ and *V*_th,0_ represent the values of the mobility and threshold voltage corrected by the effect of *R*_C_. As a consequence, the transconductance *g*_m_ = d*I*_D_/d*V*_G_ can be expressed as:

[4]
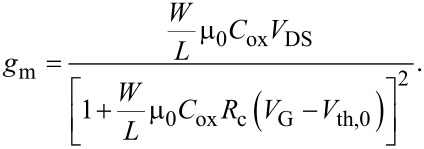


**Figure 5:**
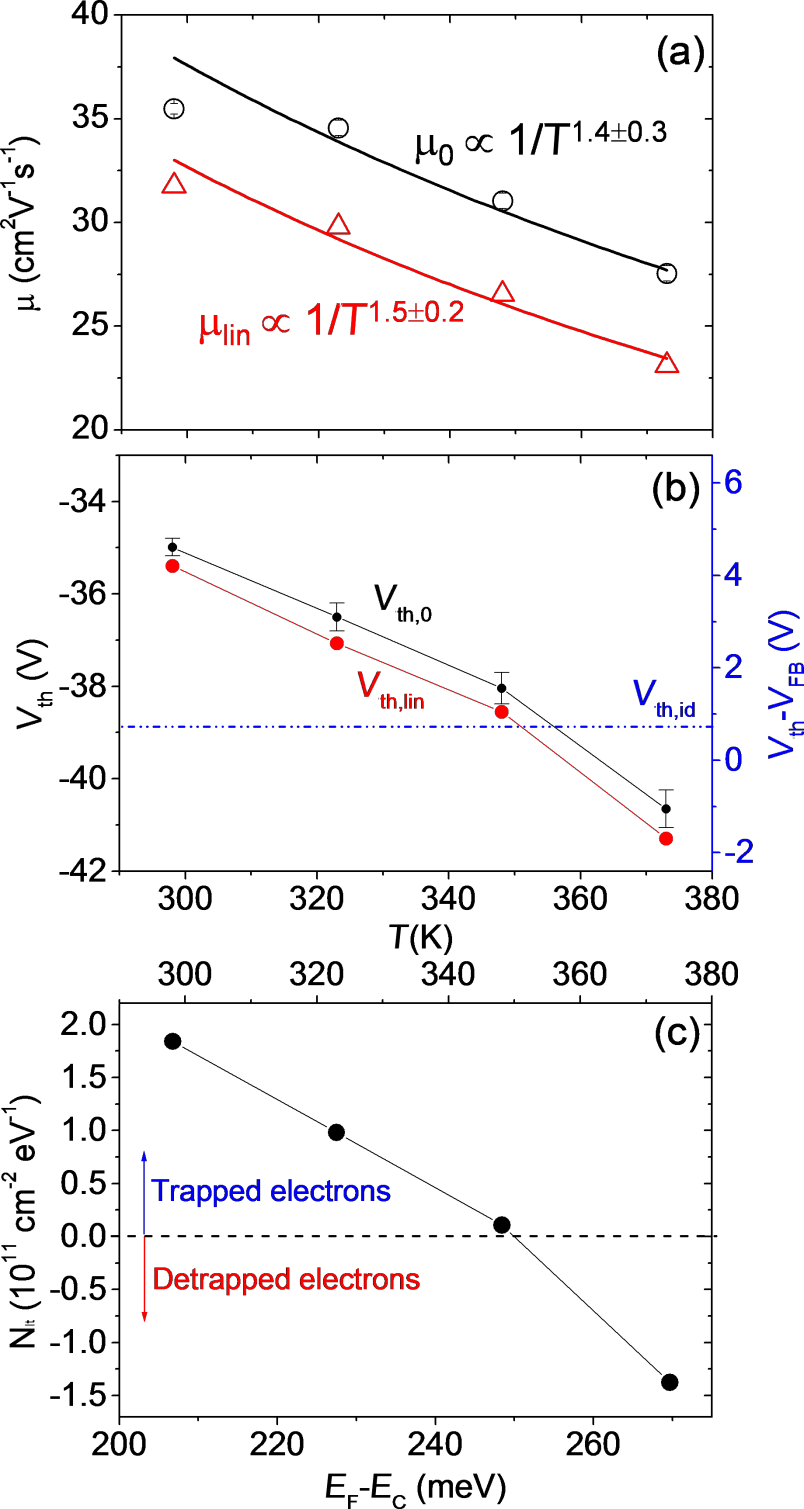
(a) Temperature dependence of the field effect mobility extracted from the linear region of the *I*_D_−*V*_G_ characteristics without (μ_lin_) and with (μ_0_) the correction for the effect of the contact resistance *R*_C_. Experimental data have been fitted with a temperature dependence 1/*T*^α^. (b) Temperature dependence of the threshold voltage evaluated without (*V*_th,lin_) and with (*V*_th,0_) the correction for the effect of the contact resistance *R*_C_. (c) Density of trapped/detrapped electrons at MoS_2_/SiO_2,_ as a function of *T* (upper scale) and the corresponding position of the Fermi level with respect to the conduction band (*E*_F_−*E*_C_).

Noteworthy, the ratio 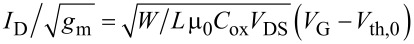 is independent of *R*_C_. A plot of *I*_D_/*g*_m_^1/2^ vs *V*_G_ is reported in Figure 4b. The corrected value of the field effect mobility (μ_0_ = 35.48 ± 0.25 cm^2^V^−1^s^−1^) can be calculated from the slope of the linear fit of these data, whereas the threshold voltage (*V*_th,0_ = −34.99 ± 0.19 V) can be obtained from the intercept with the *x* axis. It is worth noting that the mobility value μ_0_ after correction for the contact resistance is more than 10% higher than the value estimated without any correction, whereas the threshold voltage *V*_th,0_ after correction is only 1% higher than the value estimated without accounting for *R*_C_. This indicates that the underestimation of the mobility neglecting the contact resistance effect can be quite relevant, whereas the threshold voltage is less affected by *R*_C_. By repeating this procedure for all the measured characteristics reported in Figure 2b,c, the temperature dependence of the mobility (μ_lin_ and μ_0_), threshold voltage (*V*_th_ and *V*_th,0_) in the considered temperature range has been evaluated, as illustrated in Figure 5a,b, respectively.

Both μ_lin_ and μ_0_ were found to decrease as a function of *T* with a similar dependence 1/*T*^α^, with α = 1.5 ± 0.2 in the case of μ_lin_ and α = 1.4 ± 0.3 in the case of μ_0_. Such a dependence of μ ≈ 1/*T*^α^ with α > 1 indicates that the main mechanism limiting the mobility of electrons in the multilayer MoS_2_ channel in this temperature range is scattering by optical phonons, as reported by other experimental and theoretical investigations [4]. Instead, electron mobility was found to be limited by Coulomb scattering by charged impurities only at lower temperatures (<100 K) [4]. Noteworthy, scattering by charged impurities at the interface with the substrate results in the dominant mechanism for another well-studied 2D material, graphene, even at room temperature and higher temperatures [17–18].

In Figure 5b, the threshold voltage *V*_th_ exhibits a negative shift of about 6 V with increasing the temperature from 298 to 273 K. For convenience, the difference *V*_th_−*V*_FB_ is also reported in Figure 5b, right scale. It is useful to compare the experimental temperature dependence of *V*_th_ with the expected theoretical variation with temperature, in order to understand which are the relevant physical parameters ruling this behavior.

For an ideal transistor (without interface states) operating under accumulation conditions, the shift between the threshold voltage *V*_th,id_ and the flat band voltage *V*_FB,id_ can be expressed as:

[5]
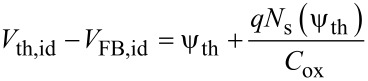


where ψ_th_ is the downward (negative) band bending at the threshold (as illustrated in the band diagram (iii) of Figure 3c, and *N*_s_(ψ_th_) is the electron density in the channel at ψ_th_

[6]
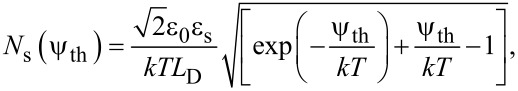


where 
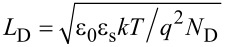
 is the Debye length. The band bending ψ_th_ can be evaluated assuming that the electron density in the channel at the threshold corresponds to *N*_s_(ψ_th_) = *tN*_D,_ where *N*_D_ is the uniform doping concentration in the MoS_2_ thin film and *t* its thickness. Assuming *N*_D_ = 10^16^ cm^−3^ for our unintentionally doped MoS_2_, we obtain *N*_s_ ≈ 4 × 10^10^ cm^−2^. Furthermore, a value of ψ_th_ ranging from approximately −34 meV (at *T* = 298 K) to −39 meV (at *T* = 373 K) can be estimated from the dependence of *N*_s_ on ψ_th_ in Equation 6. Under these assumptions, *V*_th,id_−*V*_FB,id_ ≈ 0.7 eV (nearly independent of *T*) can be estimated, as indicated in Figure 5b (blue dashed line).

In order to account for the large change of *V*_th_ with temperature, the role of interface states at SiO_2_/MoS_2_ interface must be considered. The difference between the experimental *V*_th_−*V*_FB_ and theoretical *V*_th,id_−*V*_FB,id_ can be described by a term Δ*V*_it_ = *qN*_it_/*C*_ox_, where *N*_it_ is the density of trapped/detrapped electrons by SiO_2_ interface traps. These interface traps exhibit a donor like behavior, i.e., they are positively charged above the Fermi level (when they are empty) and neutral below the Fermi level (when they are filled by electrons) [13]. Hence, electron trapping results in a neutralization of the interface states, resulting in a positive shift of *V*_th_ with respect to *V*_FB_ (i.e. Δ*V*_it_ > 0). On the contrary, detrapping of electrons from these states results in an increase of the positive charge and, hence, in Δ*V*_it_ < 0.

From the experimental data in Figure 5b, trapped electron densities *N*_it_ = 2 × 10^11^, 1 × 10^11^, and 2 × 10^10^ cm^−2^ are estimated at 298, 323, and 348 K, respectively, whereas a detrapped electron density *N*_it_ = 1.3 × 10^11^ cm^−2^ is obtained at 373 K (see Figure 5c). Electron trapping and detrapping at MoS_2_/SiO_2_ interface have been shown to be thermally activated processes [13]. Hence, for a given interface trap distribution *D*_it_ close to the MoS_2_ conduction band, *N*_it_ can be expressed as

[7]
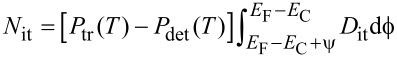


where *P*_tr_(*T*) and *P*_det_(*T*) are the trapping and detrapping probabilities, respectively [13]. The experimentally found temperature dependence of *N*_it_ can be explained as follows. As *T* increases, the shift of the Fermi energy *E*_F_ with respect to *E*_C_ increases as


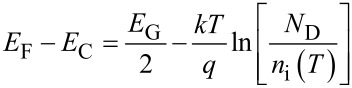


resulting in a change of the integration range in Equation 7. Furthermore, the difference *P*_tr_(*T*)−*P*_det_(*T*) can change with *T*. The dependence of *N*_it_ on *E*_F_ is also illustrated in Figure 5c. It is consistent with a decrease of *D*_it_ with increasing *E*_F_−*E*_C_. Furthermore, at 373 K, it can be argued that the *P*_det_ becomes higher than *P*_tr_, resulting in a negative value of *N*_it_.

### Output characteristics

**Figure 6:**
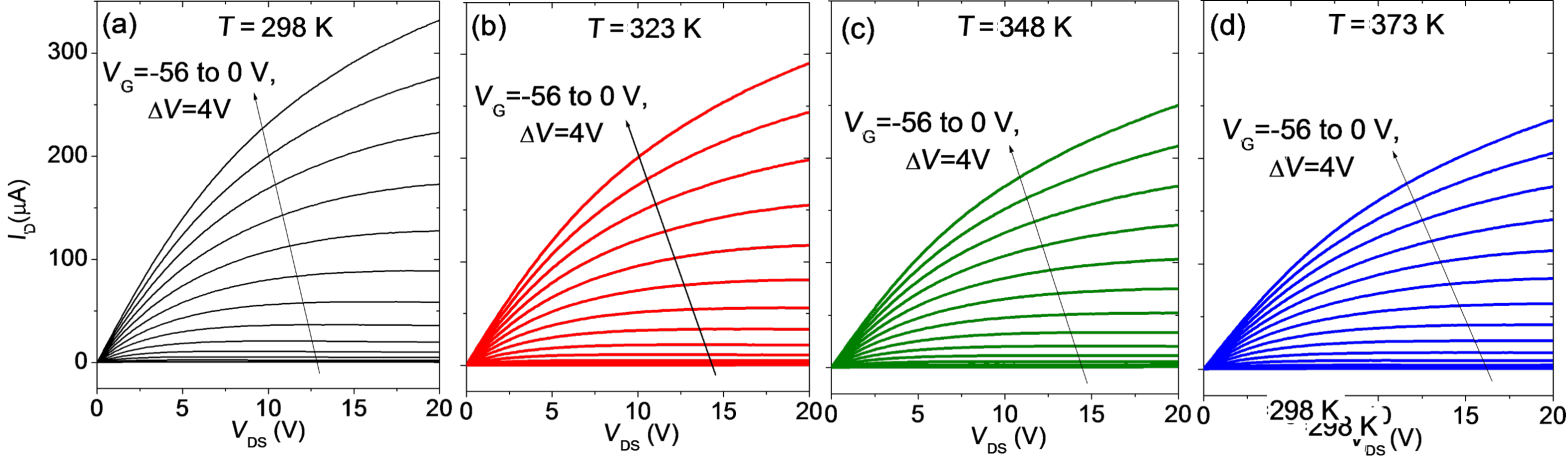
Output characteristics *I*_D_−*V*_DS_ for different gate bias values from −56 to 0 V at different temperatures: (a) *T* = 298 K, (b) *T* = 323 K, (c) *T* = 348 K and (d) *T* = 373 K.

Figure 6 shows the output characteristics *I*_D_−*V*_DS_ for different gate bias values ranging from *V*_G_ = −56 to 0 V (with steps Δ*V* = 4 V) measured at different temperatures, i.e., (a) 298 K, (b) 323 K, (c) 348 K and (d) 373 K. For all the *V*_G_ values, *I*_D_ exhibits a linear increase with *V*_DS_ at low drain bias (*V*_DS_ << *V*_G_−*V*_th_), whereas it deviates from the linear behavior at larger *V*_DS_. In particular, current saturation is achieved whenever the condition *V*_DS_ > *V*_G_−*V*_th_ is reached. By comparing the output characteristics measured at the different temperatures with the same *V*_G_ values, it is evident that both the slope of the *I*_D_−*V*_DS_ curves in the linear region and the saturation current value decreases with increasing *T*.

The reciprocal of the *I*_D_−*V*_DS_ curves slope in the linear region at low *V*_DS_ is the device on-resistance *R*_on_, which can be expressed as:

[8]
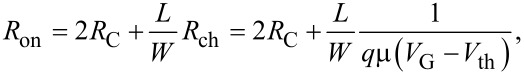


where *R*_C_ is the source and drain contact resistance and *R*_ch_ the channel sheet resistance, which depends inversely on (*V*_G_−*V*_th_), according to Equation 2.

**Figure 7:**
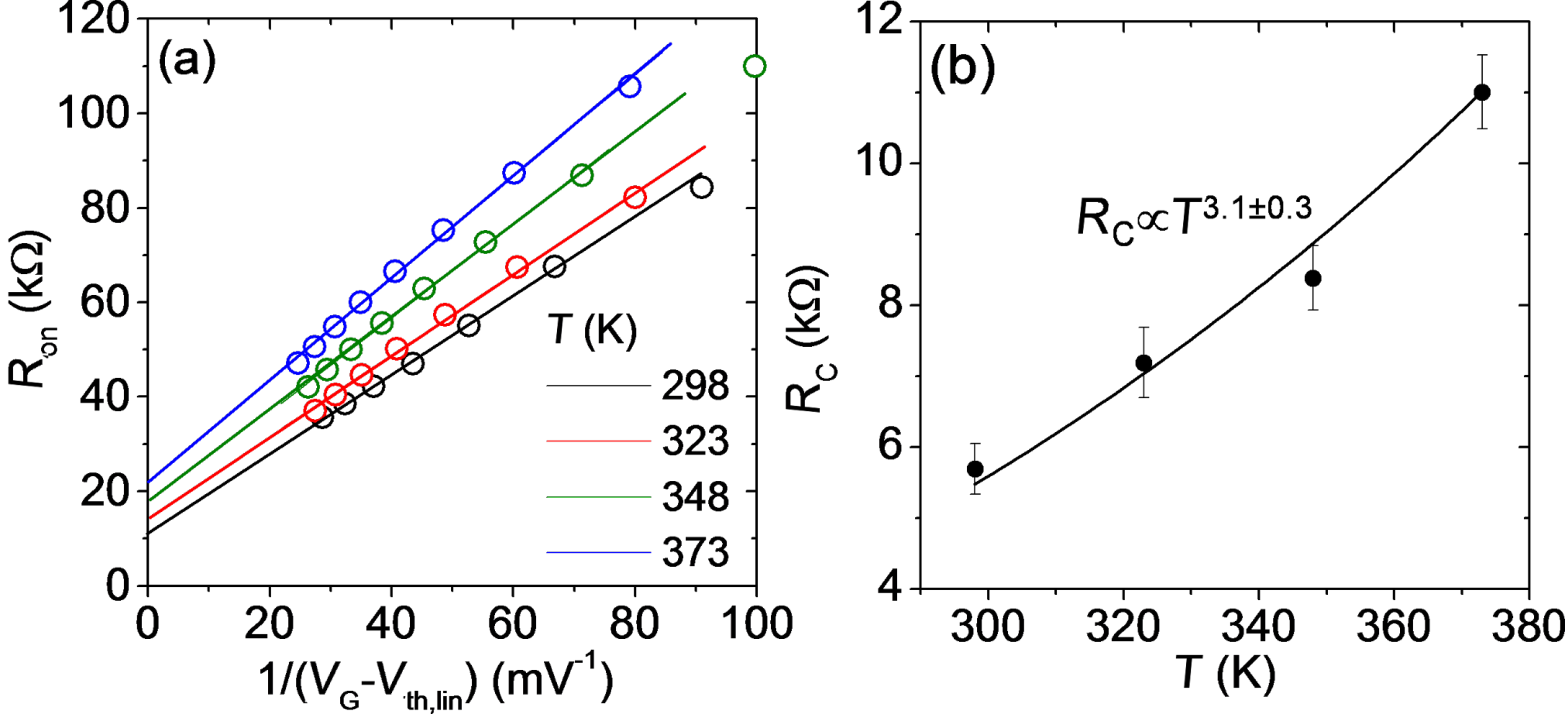
(a) On-resistance *R*_on_ vs 1/(*V*_G_−*V*_th,lin_) at different temperatures. (b) Temperature dependence of *R*_C_.

Figure 7a reports the plots of *R*_on_ vs 1/(*V*_G_−*V*_th,lin_) extracted from the *I*_D_−*V*_DS_ characteristics in Figure 6 at the different temperatures. The linear fit of the data was performed for the four temperatures and, from the intercept with the vertical axis, the value of the contact resistance *R*_C_ was estimated. The behavior of *R*_C_ vs T is reported in Figure 7b, indicating an increase of *R*_C_ 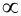 *T*^α^, with α = 3.1 ± 0.3.

**Figure 8:**
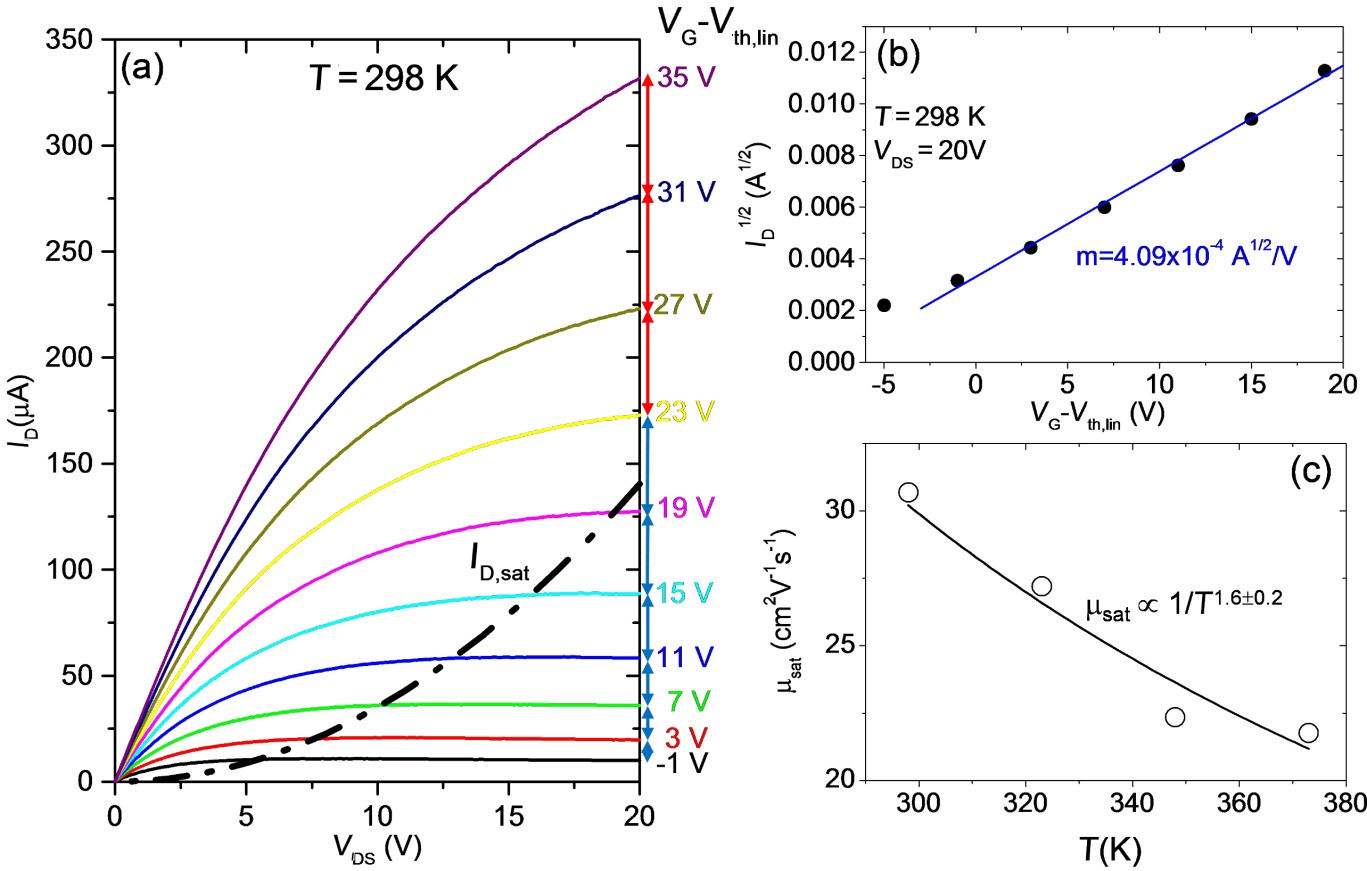
(a) Output characteristics (*I*_D_−*V*_DS_) for different gate bias values from −56 to 0 V at *T* = 298 K. (b) Plot of *I*_D_^1/2^ vs *V*_G_−*V*_th,lin_ for *V*_DS_ = 20 V and linear fit of the data. (c) Saturation mobility μ_sat_ vs temperature.

Finally, the behavior of the output characteristics at high *V*_DS_ is discussed. Figure 8a shows the *I*_D_−*V*_DS_ characteristics measured at *T* = 298 K. The *V*_G_−*V*_th_ value for each curve is indicated. It can be observed that the current saturation regime (i.e., *I*_DS_ independent of *V*_DS_) is reached only for *V*_G_−*V*_th_ < 20 V, corresponding to an accumulated electron density in the channel *N*_s_ < 1.1 × 10^12^ cm^−2^. For *V*_G_−*V*_th_ > 20 V, saturation is not reached.

In the saturation condition, *I*_D_ is only a quadratic function of *V*_G_−*V*_th_ [14]:

[9]
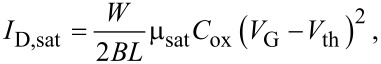


where μ_sat_ is the mobility value under saturation conditions and the term *B* is the so-called body coefficient, which depends on the gate oxide capacitance, on the doping concentration in the film and on the temperature:


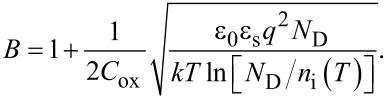


For thin gate dielectrics and low doping in the film, *B* can be approximated to 1, but for thick dielectrics and high doping its value can be significantly higher. In the case of our device with *C*_ox_ = 9.1 × 10^−5^ F/m and assuming a MoS_2_ doping *N*_D_ ≈ 1 × 10^16^ cm^−3^, *B* can range from ≈2.97 to ≈3.12 in the considered temperature range. In Figure 8b, *I*_D_^1/2^ at *V*_DS_ = 20 V is reported as a function of *V*_G_−*V*_th_, showing a linear behavior. According to Equation 9, the mobility under saturation condition can be evaluated from the slope *m* of the fit, as 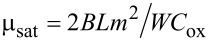. By repeating this procedure for all the output characteristics measured at the different temperatures, the behavior of μ_sat_ as a function of *T* can be obtained. The main error source in the estimation of μ_sat_ is related to the fact that the doping concentration *N*_D_ and, hence, the coefficient *B* is not exactly known. Noteworthy, the values of μ_sat_ in Figure 8c, estimated assuming N_D_ ≈ 1 × 10^16^ cm^−3^, are very close to those evaluated from the linear region of the transfer characteristics (see Figure 5a) and exhibit a similar temperature dependence. This also confirms that the assumption for the doping concentration is correct.

## Conclusion

In conclusion, a temperature dependent investigation of back-gated multilayer MoS_2_ transistors with Ni source/drain contacts in the range from *T* = 298 to 373 K has been performed. The SBH Φ_B_ ≈ 0.18 eV of the Ni/MoS_2_ contact was evaluated from the analysis of the transfer characteristics *I*_D_−*V*_G_ in the subthreshold regime. The resulting *R*_C_ associated with the SBH was determined by fitting the *R*_on_ dependence on 1/(*V*_G_−*V*_th_) extracted from the device output characteristics *I*_D_−*V*_DS_ at low *V*_DS_. An increase of *R*_C_ 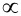 *T*^3.1^ was demonstrated. The impact of *R*_C_ on the values of μ and *V*_th_ values was determined, showing an underestimation of μ by more than 10% if the effect of *R*_C_ is neglected, whereas the influence of *R*_C_ on the estimated value of *V*_th_ is only 1%. Furthermore, the temperature dependence of μ and *V*_th_ was investigated, showing a decrease of μ ≈ 1/*T*^α^ with α = 1.4 ± 0.3 (indicating scattering by optical phonons as the limiting mechanism), and a negative shift of *V*_th_ by about 6 V with increasing *T*. The role played by electron trapping at the MoS_2_/SiO_2_ interface to explain such a large *V*_th_ shift was discussed.

## Experimental

Back-gated transistors were fabricated using MoS_2_ flakes exfoliated from molybdenite bulk crystals (supplier SPI [19]) with thicknesses ranging from ≈40 to ≈50 nm and transferred onto a highly doped n-type Si substrate covered with 380 nm of thermally grown SiO_2_. An accurate sample preparation protocol has been adopted for controlled quality of the MoS_2_/SiO_2_ interface, as this is crucial to achieve reproducible electrical behavior of the devices. In particular, thermo-compression printing using a Karl-Suss nanoimprint device with fixed temperature and pressure conditions [20–21] has been employed to transfer the exfoliated MoS_2_ flakes onto the SiO_2_ surface that was previously cleaned using solvents and a soft O_2_ plasma treatment. Finally, source and drain contacts were obtained by deposition and lift-off of a Ni(50 nm)/Au(100 nm) bilayer.

The temperature-dependent electrical characterization in the range from 298 to 373 K was performed using a Cascade Microtech probe station with an Agilent 4156b parameter analyzer. All the measurements were carried out in dark conditions and under nitrogen flux.
